# Psychosocial Problems in the Siblings of Multitransfused Thalassemia Patients

**DOI:** 10.7759/cureus.86443

**Published:** 2025-06-20

**Authors:** Pratibha Lodhi, Shweta Sharma, Prachi Singh, Amit Agrawal

**Affiliations:** 1 Department of Pediatrics, Bundelkhand Medical College, Sagar, IND; 2 Pediatrics, Gandhi Medical College, Bhopal, IND; 3 Pediatrics and Neonatology, All India Institute of Medical Sciences, Bhopal, Bhopal, IND

**Keywords:** anxiety, beta-thalassemia, depression, psychological stress, siblings

## Abstract

Background: Thalassemia significantly affects the psychosocial health of patients, their families, and siblings. Recognizing and addressing psychosocial challenges within the family system and among siblings is crucial to enhance their well-being and the overall family functioning.

Objectives: To assess the psychosocial stress in siblings of multiple-transfused thalassemia patients, utilizing two distinct scales specifically designed to evaluate psychological distress in children.

Methodology: In this cross-sectional observational study, siblings aged 2-18 years accompanying beta-thalassemia patients attending the Pediatrics Department of Gandhi Medical College, Bhopal, for blood transfusions or outpatient department visits were enrolled. Psychosocial stress was assessed using the Pediatric Symptom Checklist-17 (PSC-17) for the participants aged 4-17 years and the Perceived Stress Scale - Children (PSS-C) for the participants aged 5-18 years. Regression, latent class, and mediation analyses explored sociodemographic associations, symptom profiles, and PSS-C as a mediator of PSC-17 outcomes.

Results: Of 95 participants, 71 completed the PSS-C, with 38 (53.5%) reporting moderate stress and 33 (46.5%) low stress. On PSC-17 screening, 30 (31.6%) screened positive for internalising symptoms, and 9 (9.5%) each for attention and externalizing problems. Older age was associated with higher odds of internalizing concerns, and male gender with lower odds of attention deficit hyperactivity disorder (ADHD) and externalizing symptoms. On latent class analysis, a 2-class model demonstrated the best fit based on the lowest Bayesian information criterion (249.51). Class 1 represented a high-symptom group, characterized by a high probability of screening positive across all three domains, whereas Class 2 represented a low-symptom group showing low probabilities of concern. Mediation analyses revealed no significant indirect effects of perceived stress (PSS-C score) on the relationship between demographic characteristics and psychosocial symptoms across all PSC-17 domains.

Conclusion: We found that siblings of thalassemia patients experience mild to moderate levels of psychological stress. Anxiety and depression emerged as the most prevalent disorders, with a higher incidence observed in females than in males. It advocates for a more inclusive, holistic approach that considers both the patient and the psychosocial well-being of siblings within the broader family context.

## Introduction

Thalassemia is an inherited blood disorder caused by mutations in the α (HBA1/HBA2) or β (HBB) globin genes, typically following an autosomal recessive pattern. These genetic defects impair the production of hemoglobin A (HbA), a molecule composed of two α and two β globin chains [1]. In 2021, the global incidence of thalassemia was estimated at 119,679 cases, accounting for approximately 13.1 million cases worldwide. However, these figures carry considerable uncertainty, as reflected by the broad uncertainty intervals ranging from 93,218 to 153,985 for incidence and 1.1 to 1.57 million for total cases, highlighting the need for cautious interpretation [2]. India bears a significant thalassemic burden, contributing nearly 25% to the global β-thalassemia cases, with an estimated 100,000 diagnosed patients. However, the true prevalence remains unknown due to the lack of a national patient registry [3].

Non-compliance with the treatment due to psychosocial factors remains the primary cause of death in children in developing countries [4]. Findings indicated that the greatest psychosocial burden occurred during adolescence, a period characterised by critical developmental tasks such as identity formation, establishing intimate relationships, and introduction to the working environment [5]. Various physical, emotional, and cognitive disturbances disrupt the quality of life. Therefore, incorporating and accepting thalassemia into a child's self-concept is particularly difficult during adolescence [6].

Thalassemia not only affects the psychological health of patients but also their families and siblings. The main factors contributing to psychological problems among patients with thalassemia may be related to the disease itself (chronic nature, hospitalization, school absenteeism, blood transfusion, iron chelation), family (negligent parents, overprotective parents, hostile parents), and social factors (uncompassionate peers) [7]. It is essential to identify and assess psychological issues in families and siblings involved in the care of children with thalassemia. However, in India, the psychosocial aspect of the family as a unit is often neglected, and medical management of patients with thalassemia and its complications is the only management strategy to prolong the life of the patient. Residents of smaller cities often find it more difficult to access mental health support, as these communities typically have fewer resources than larger cities [8]. The psychosocial burden experienced by children and adolescents with thalassemia significantly impacts numerous aspects of their lives, including education, school absenteeism, participation in sports, perceived differences from friends and siblings, social interactions, family adjustment, anxiety levels, feelings of isolation, and experiences of stigmatization [9]. It also affects the economic status of the family. Research has indicated that individuals with thalassemia often encounter challenges in their relationships with siblings and peers, potentially hindering the development of comprehensive interpersonal connections. Similarly, children with thalassemia who experienced complications reported higher levels of perceived difference from their friends and siblings compared with those who did not experience such complications [10,11].

Although many studies have sought to elucidate the impact of chronic health conditions (CHCs) on the psychosocial functioning of siblings, the findings have often been inconsistent. However, a systematic review conducted by Vermaes et al. identified a significant negative effect of CHCs on siblings, rendering them vulnerable to internalizing problems characterized by highly intrusive thoughts and a notable risk of psychological difficulties [12]. A study by Al Kloub et al. reported stronger family relationships in families affected by thalassemia compared to control groups [13]. However, their analysis also indicated that psychological impairment within these families was associated with factors such as low family income, larger family size, and the presence of siblings with thalassemia [13]. Considering the identified gap in the existing literature, the present study was designed to assess the psychosocial stress experienced by siblings of patients undergoing multiple blood transfusions for thalassemia.

## Materials and methods

The present study was a descriptive cross-sectional study conducted among siblings of multi-transfused thalassemia patients to assess their psychosocial problems. This study was conducted from 15 September 2021 to 14 September 2022 in the Department of Pediatrics, Gandhi Medical College, Bhopal. Two to 18-year-old siblings of thalassemia patients were included in the study. Screening of the siblings for thalassemia was conducted, and siblings reporting positive results were excluded from the study. Siblings with a known psychological disorder, any other chronic diseases, such as congenital heart disease, chronic liver failure, chronic kidney disease, or any malignancies, and those requiring regular blood transfusions were excluded. Prior approval was obtained from the institutional ethics committee before recruiting the patients. 

We screened siblings of all thalassemia children registered in the thalassemia day care center according to the inclusion and exclusion criteria. All siblings of consecutively admitted thalassemia patients who fulfilled the inclusion criteria were recruited in the study after getting informed consent from the parents or legal guardians. Based on this, the present study included 95 participants aged between two and 18 years who were siblings of individuals diagnosed with beta-thalassemia. Sociodemographic details of the siblings and the family history were recorded. Following this, the psychosocial status of the included participants was assessed using two scales: the Pediatric Symptom Checklist-17 (PSC-17) and Perceived Stress Scale for Children (PSS-C), both in their regional language (Hindi) and English [14,15]. At-risk patients were referred to the psychiatry department for further evaluation and management.

The PSC-17 is a behavioral screening questionnaire that consists of 17 items, grouped into three categories: Internalizing (5 items), Attention (5 items), and Externalizing (7 items). Each question is answered using a scale where 0 means "Never," 1 means "Sometimes," and 2 means "Often." To identify specific behavioral concerns, a score of 5 or higher on the Internalizing section suggests anxiety or depression, a score of 7 or higher on the Attention section points to possible attention deficit hyperactivity disorder (ADHD), and a score of 7 or higher on the Externalizing section indicates externalizing behaviors. A total score of 15 or above suggests the presence of significant behavioral or emotional difficulties [14].

The Perceived Stress Scale for Children (PSS-C) was utilized to assess stress levels. This self-report instrument comprises 13 items, each rated on a 4-point Likert scale ranging from ‘never’ to ‘a lot’ (‘never’, ‘little’, ‘sometimes’, ‘a lot’). The items evaluate perceived stress related to time pressure, academic challenges, and relationships with family and peers. Total score ranges from 1 to 39, with higher scores reflecting greater perceived stress (1-13: low stress; 14-26: moderate stress; 27-39: high stress). Importantly, the PSS-C emphasizes the child’s subjective experience of stress rather than objective stressors [15]. The PSS-C has been used globally, and this valuable tool is freely available for research and non-commercial applications. Similarly, PSC-17 can be freely reproduced for research purposes [15].

Data obtained were entered in Microsoft Excel and then statistically analysed using the Statistical Package for Social Sciences (IBM SPSS software version 20) and R Studio Version 2024.12.1+563. Categorical data were described as frequency and proportion, whereas continuous variables were expressed as mean and standard deviation. The statistical test carried out was the Chi-square (χ2) test; however, Fisher’s Exact Test was used when expected cell counts were less than 5, and a p-value less than 0.05 was considered statistically significant. The causal mediation analysis was assessed with the help of RStudio (Posit | The Open-Source Data Science Company).

Multivariable logistic regression models were conducted to examine the associations between sociodemographic variables and four domains of psychosocial concerns measured by the PSC-17 scale and perceived stress levels derived from the PSS-C, categorized into low and moderate stress. Unadjusted models included age and gender, while adjusted models additionally controlled for religion, region (urban/ rural), parental marital status, BMI classification, and High-Performance Liquid Chromatography (HPLC) screening status where applicable. Odds ratios (ORs) and adjusted odds ratios (aORs) with 95% confidence intervals (CIs) and associated p-values were reported. Analyses were conducted using complete-case methodology, excluding observations with missing covariate data.

Latent class analysis (LCA) was conducted to identify subgroups of psychosocial symptom patterns based on PSC-17 domains. Each domain was recoded as a binary categorical variable (1 = Absent, 2 = Present). Models with two to four classes were estimated using the expectation-maximization algorithm, and model fit was compared using the Bayesian Information Criterion (BIC), with the two-class model showing the best fit. Class profiles were interpreted using conditional response probabilities, and individuals were assigned to classes based on their highest posterior probability.

Causal mediation analysis was performed. The mediator model was fitted using linear regression, while outcome models were estimated using logistic regression for each binary psychosocial domain. Nonparametric bootstrapping with 1,000 simulations was applied to estimate the average causal mediation effect (ACME), average direct effect (ADE), total effect, and the proportion mediated [16,17]. Female siblings and those under five years were set as control groups, reflecting the reference categories within the cohort. Comparisons were made against male siblings and those aged 11-15 years to examine whether perceived stress mediated observed differences in psychosocial outcomes across key developmental and demographic contrasts.

## Results

The present study screened 95 siblings of 61 thalassemia patients for psychosocial problems, with almost equal numbers of females and males (48 and 47, respectively). Only four (4.2%) siblings were born to parents with a history of consanguineous marriage. HPLC is the most sought-after method to diagnose blood-related dyscrasias in thalassemia patients. However, HPLC was not done in most siblings (81, 85.3%), while the remaining had a normal HPLC value (11, 11.6%), and thalassemic trait in three (3.2%) siblings. Similarly, antenatal testing was not done for 93 (97.9%) siblings, while 2 (2.1%) had a heterozygous trait (Table 1).

**Table 1:** Sociodemographic details of the study participants

Variables		Frequency		Percentage
Number of siblings (n=61)				
1		21		34.4
2		25		41.0
3		13		21.3
4		1		1.6
5		1		1.6
Age of sibling (years) (n=95)				
<5		24		25.3
6-10		30		31.6
11111-15		33		34.7
>15		8		8.4
Sex (n=95)				
Male		47		49.5
Female		48		50.5
Anthropometry – Weight for height (≤5 years)				
3		12.5	
-3 to -2 SD	2		8.3	
-2 to -1 SD	10		41.7	
-1 SD to Median	6		25.0	
Median to +1SD	2		8.3	
+1 to +2 SD	1		4.2	
Anthropometry – Body Mass Index (>5 years)				
<3^rd^ Centile	4		5.6	
3^rd^ to 10^th^ Centile	24		33.8	
10^th^ to 50^th^ centile	34		47.9	
>50^th^ centile	9		12.7	
Consanguinity				
No	91		95.8	
Yes	4		4.2	
Religion				
Hindu	66		69.5	
Muslim	29		30.5	
Residence				
Rural	57		60.0	
Urban	38		40.0	
High-Performance Liquid Chromatology				
Normal	11		11.6	
Trait	3		3.2	
Not done	81		85.3	

Perceived stress levels were predominantly moderate, reported by 38 (53.5%) participants, while 33 (46.5%) reported low stress levels. There was no difference in the stress levels between males and females (p = 0.63) or between different age groups (p = 0.78) (Table 2).

**Table 2:** Stess levels in siblings of thalassemia patients by Perceived Stress Scale for Children (PSS-C) (n=71) Statistical analysis performed using Fisher’s Exact Test. Data are presented for 71 out of 95 participants due to missing responses on the PSS-C scale.

Variables	Categories	Perceived Stress Levels			P value
		Low	Moderate	High	
Age (year)	6-10	15 (50)	15 (50)	0	0.78
	11-15	14 (42.4)	19 (57.6)	0	
	>15	4 (50)	4(50)	0	
Gender	Male	17 (50)	17 (50)	0	0.63
	Female	16 (43.2)	21 (56.8)	0	
Total		33 (46.5)	38 (53.5)	0	

The emotional and behavioral symptoms among siblings were evaluated using the PSC-17 checklist. Analysis of the responses revealed that, except for a small subset (nine individuals, 9.5%), most siblings did not exhibit symptoms indicative of attention deficit hyperactivity disorder (ADHD) or other externalizing disorders. Additionally, anxiety and depression were found to be absent at similar rates among both males and females (p = 0.82). However, symptoms of ADHD were identified more frequently in female siblings compared to males (p = 0.03), according to the checklist scores (Table 3). Age group assessment revealed that all the siblings above 15 years had no psychosocial problems, and these were similar across all the age groups except for anxiety and depression, which were more common in children aged 5-10 years and 10-15 years (p = 0.02) (Table 3).

**Table 3:** Psychosocial issues identified by the Pediatric Symptom Checklist-17 in siblings of thalassemia patients Chi-square test was performed for group comparisons; NA – Not applicable (where Fisher’s Exact Test was used because the expected cell counts were less than 5). A p-value less than 0.05 is considered statistically significant. (%) represents the frequency of distribution.

Variables	Category				
		Absent, n (%)	Present, n (%)	Chi-Square value	p value
Total		65 (68.4)	30 (31.6)		
Anxiety and depression					
Gender	Male	33 (70.2)	14 (29.8)	0.02	0.82
	Female	32 (66.7)	16 (33.3)		
Age	<5	20 (83.3)	4 (16.7)	NA	0.02
	5 -10	18 (60)	12 (40)		
	10 - 15	19 (57.6)	14 (42.4)		
	>15	8 (100)	0		
Attention-deficit hyperactivity disorder					
Gender	Male	46 (97.9)	1 (2.1)	4.28	0.03
	Female	40 (83.3)	8 (16.7)		
Age	<5	22 (91.7)	2 (8.3)	NA	0.88
	5 -10	26 (86.7)	4 (13.3)		
	10 - 15	30 (90.9)	3 (9.1)		
	>15	8 (100)	0		
Externalizing					
Gender	Male	45 (95.7)	2 (4.3)	1.8	0.15
	Female	41 (85.4)	7 (14.6)		
Age	<5	22 (91.7)	2 (8.3)	NA	0.88
	5 -10	26 (86.7)	4 (13.3)		
	10 - 15	30 (90.9)	3 (9.1)		
	>15	8 (100)	0		
Any					
Gender	Male	39 (83)	8 (17)	1.3	0.22
	Female	34 (70.8)	14 (29.2)		
Age	<5	20 (83.3)	4 (16.7)	NA	0.26
	5 -10	22 (73.3)	8 (26.7)		
	10 - 15	23 (69.7)	10 (30.3)		
	>15	8 (100)	0		

Table 4 presents unadjusted and aORs for predictors across PSC-17 domains. Male siblings had significantly lower odds of screening positive for ADHD in the unadjusted model, with this association nearing significance after adjustment. A similar trend was noted for externalizing problems. Siblings aged 6-10 and 11-15 years had a significantly higher aOR of anxiety and depression compared to those under 5 years, though no age group was significantly associated with ADHD, externalizing, or the overall "Any" domain. Estimates for the >15 years group were near-zero across models, indicating quasi-complete separation.

**Table 4:** Logistic regression of Pediatric Symptom Checklist-17 (PSC-17) domains OR - Odds Ratio, NA- Not applicable. A p-value less than 0.05 is considered statistically significant.

Predictors	Psychosocial Concerns					
	OR (Unadjusted)	95 % CI (Unadjusted)	p value (Unadjusted)	OR (Adjusted)	95 % CI (Adjusted)	p value (Adjusted)
Anxiety and Depression						
Gender (Male vs. Female)	0.9	0.36–2.27	0.82	0.97	0.34–2.73	0.95
Age 6–10 vs. <5 years	3.34	0.97–13.71	0.06	5.11	1.30–26.1	0.02
Age 11–15 vs. <5 years	3.63	1.07–14.70	0.049	5.19	1.33–26.7	0.02
Age >15 vs. <5 years	1.17 × 10⁻⁷	5.26×10⁻¹⁸¹–7.71×10²³	0.99	1.59 × 10⁻⁷	1.91×10⁻¹⁹¹–1.38×10²¹	0.99
Attention-deficit hyperactivity disorder						
Gender (Male vs. Female)	0.10	0.005–0.58	0.03	0.1	0.00–0.71	0.05
Age 6–10 vs. <5 years	1.88	0.31–15.44	0.509	4.07	0.47–90.17	0.25
Age 11–15 vs. <5 years	0.85	0.12–7.19	0.87	1.98	0.20–44.81	0.58
Age >15 vs. <5 years	0	NA–7.25×10⁴⁸	0.99	0	NA–7.32×10¹⁰⁸	0.99
Externalising						
Gender (Male vs. Female)	0.24	0.03–1.08	0.08	0.16	0.01–0.96	0.07
Age 6–10 vs. <5 years	1.8	0.31–14.3	0.53	1.76	0.29–14.75	0.55
Age 11–15 vs. <5 years	0.91	0.14–7.58	0.92	0.79	0.11–7.00	0.82
Age >15 vs. <5 years	0	NA–6.68×10⁷³	0.99	0	NA–1.54×10⁷⁴	0.99
Any						
Gender (Male vs. Female)	0.51	0.18–1.37	0.18	0.59	0.18–1.77	0.35
Age 6–10 vs. <5 years	1.87	0.50–8.01	0.36	2.66	0.64–14.03	0.20
Age 11–15 vs. <5 years	2.0	0.56–8.29	0.3	2.5	0.62–12.93	0.22
Age >15 vs. <5 years	0	NA–3.01×10²⁷	0.99	0	0–3.06×10³⁷	0.99

In the binary logistic regression (moderate versus low perceived stress levels), no demographic factors emerged as statistically significant predictors. Compared to children aged six to 10 years, neither older age groups (11-15, >15), nor gender, region, religion, or BMI status showed significant associations with elevated stress (all p > 0.5). The under five years age group was excluded due to the absence of cases in either stress category (Table 5).

**Table 5:** Logistic regression of perceived stress OR - Odds ratio, CI - Confidence level, BMI - Body Mass Index. A p-value less than 0.05 is considered statistically significant.

Predictor	OR (Unadjusted)	95 % CI (Unadjusted)	p value (Unadjusted)	OR (Adjusted)	95 % CI (Adjusted)	p value (Adjusted)
Gender (Male vs. Female)	0.76	0.30–1.94	0.57	0.91	0.30–2.69	0.86
Age 11–15 vs. 6–10	1.36	0.50–3.71	0.55	1.24	0.40–3.92	0.71
Age >15 vs. 6–10	1	0.20–4.96	1.00	0.99	0.18–5.55	0.99
Region (Urban vs. Rural)	1.06	0.41–2.79	0.90	1.16	0.39–3.50	0.79
Religion (Muslim vs. Hindu)	0.95	0.33–2.77	0.93	1.17	0.33–4.31	0.81
BMI below average vs average	0.85	0.29–2.50	0.77	0.8	0.25–2.56	0.71
BMI above average vs. average	0.62	0.13–2.77	0.53	0.85	0.16–4.57	0.84

On LCA analysis, a 2-class model demonstrated the best fit based on the lowest BIC (BIC = 249.51) and was retained for interpretation. Class 1 (12% of the sample) was characterized by a high probability of screening positive across all three domains, whereas Class 2 (88%) showed low probabilities of concern. Models with 3 or 4 classes exhibited higher BIC values and estimation issues such as negative degrees of freedom or failed convergence, suggesting overfitting (see Appendices for LCA model comparison table).

Mediation analyses revealed no significant indirect effects of perceived stress (PSS-C score) on the relationship between demographic characteristics and psychosocial symptoms across all PSC-17 domains. For the age group, the ACME was non-significant across anxiety and depression, ADHD, externalizing problems, and overall symptom burden (all p >0.60), with negligible proportions of the total effect mediated through perceived stress. For gender, perceived stress did not significantly mediate the association with any PSC-17 domains (ACME p >0.80 in all models). However, direct effects of gender were observed: male siblings had significantly lower odds of screening positive for ADHD (ADE = -0.189, 95% CI: -0.333 to -0.06, p <0.001) and externalizing symptoms (ADE = -0.128, 95% CI: -0.281 to 0.00, p = 0.046), independent of perceived stress. No direct or mediated effects were significant for the anxiety and depression or “any” domains (see Appendices for mediation analysis table).

## Discussion

In this study, we included siblings of thalasemic children aged between two and 18 years, with a mean age of 9.32 ± 4.5 years. Most participants were aged 11 to 15 years (34.7%) and six to 10 years (31.6%). This was like a study that reported the mean age of 10.80±3.70 years for thalassaemic siblings [18]. The gender distribution was nearly identical, indicating no significant sex-based disparity among.

Research has consistently demonstrated that patients with thalassemia experience a range of psychosocial difficulties, including diminished self-esteem [19,20], behavioral problems [21,22], mood disorders [23,24], and psychotic symptoms [25]. These challenges may be exacerbated by factors such as frequent hospitalizations and the fear of mortality, among other associated stressors. However, the psychological impact on their families and siblings has been comparatively overlooked for many years. Parents and other family members often endure psychological distress, physical burnout, financial strain, social ramifications, and a significant need for empathy and social support [26].

White BP established the PSS-C as a straightforward and practical instrument for evaluating stress in children. Their research underscored its utility in differentiating children with anxiety or stress-related disorders [15]. The observed variations in the prevalence of psychosocial problems across different studies may be attributable to differences in study populations, the heterogeneity of reasons for admission, the utilization of diverse diagnostic tools for psychosocial stress, and other potentially confounding variables.

Sen et al. evaluated the mental health of 46 thalassemia patients and 92 of their siblings using PSC-17. Their assessment revealed that 43% of the patients screened positive for internalizing disorders (anxiety or mood-related), with a further 15% of these patients also screening positive for externalizing disorders (conduct, oppositional defiant, adjustment, or mixed disturbed mood or conduct). In contrast, the majority of siblings (92%) showed no mental health issues, with only 7.6% screening positive for externalizing disorders [27].

Our study yielded a mean PSS-C score of 14.5 ± 4.7, indicating moderate overall perceived stress. The distribution of anxiety and depression, as reflected by perceived stress levels, showed that 38 (53.5%) of the participants experienced moderate stress, while 33 (46.5%) reported low stress. Furthermore, the majority of siblings did not exhibit significant anxiety, stress, ADHD, or externalizing symptoms. These results are comparable to those of Ghasemi et al., who observed social psychological problems in 22 (37.3%) of thalassemic children, 12 (20.3%) of siblings, and 49 (20.8%) of controls [18].

In a systematic review, Vermeas et al. provided important insights into the psychological impact of chronic health conditions (CHCs) on siblings. Their analysis demonstrated a statistically small but significant negative effect, showing that siblings of children with CHCs were more likely to experience both internalizing and externalizing problems, as well as report fewer positive self-attributes when compared to control groups. Notably, the review highlighted that older siblings and those whose brothers or sisters had life-threatening or highly intrusive CHCs were particularly vulnerable to psychological challenges. In contrast, most siblings in the present study did not report significant internalizing, externalizing, or ADHD-related symptoms. This difference can be due to the small sample size, different study populations, and possible bias in our study.

This study highlights the often-neglected psychological vulnerabilities of siblings of children with thalassemia. These individuals are susceptible to moderate perceived stress, internalizing issues (anxiety, depression), externalizing behaviors, and ADHD. Age and gender appear to influence these outcomes, with younger siblings reporting more stress, older siblings more anxiety and depression, and female siblings more attention-related difficulties. This study is among the few to assess psychological distress in siblings of thalassemia patients. The PSC-17 and PSS-C scales showed no significant distress in siblings, though this should not be disregarded. Future research should compare stress levels between patients and siblings to understand the disease's impact from both viewpoints.

This study has several notable limitations. A small sample size and possible sampling bias may influence how the results are interpreted. Some of our regression models demonstrated quasi-complete separation, particularly for the >15-year age group, resulting in near-zero or unstable odds ratios, due to limited sample size; therefore, results should be interpreted with caution. Additionally, the cross-sectional nature of the research and inconsistencies in managing missing data may limit the generalizability of the findings. The application of different diagnostic tools for psychological stress, combined with varying prevalence rates of psychosocial issues within a diverse study population, further restricts the scope of the study’s conclusions. Future research should utilize larger sample sizes to enhance the generalizability of the findings. Additionally, the development or adoption of standardized assessment tools for stress and other behavioural disorders is recommended to improve the validity and reliability of future studies.

## Conclusions

The findings of this study indicate that siblings of thalassemia patients experience mild to moderate levels of psychological stress. Anxiety and depression emerged as the most prevalent disorders, with a higher incidence observed in females than in males. These psychological issues were also more common among children over the age of five, regardless of gender. These results highlight the importance of raising awareness about psychological screening, nutritional monitoring, and providing targeted counselling for siblings. Early identification, timely intervention, and appropriate support can significantly improve psychological outcomes in this population.

## Appendices

**Table 6:** LCA model comparison (PSC-17) AIC: Akaike information criterion; BIC: Bayesian information criterion.

Model	No. of Classes	No. of Parameters	Log-Likelihood	AIC	BIC	Residual Degrees of Freedom
2-Class	2	7	-108.82	231.63	249.51	0
3-Class	3	11	-108.75	239.5	267.6	-4
4-Class	4	15	-108.75	247.5	285.81	-8

**Table 7:** Mediation analysis ACME: Average causal mediation effect; ADE: Average direct effect; ADHD: Attention deficit hyperactivity disorder

Predictor	Outcome	ACME (95% CI), p-value	ADE (95% CI), p-value	Total Effect (95% CI), p-value	Prop. Mediated, p-value
Age Group	Anxiety and Depression	0.013 (–0.038 to 0.11), p=0.64	0.013 (–0.241 to 0.26), p=0.94	0.026 (–0.229 to 0.28), p=0.82	0.51, p=0.82
	ADHD	0.0002 (–0.016 to 0.02), p=0.98	–0.043 (–0.203 to 0.12), p=0.61	–0.042 (–0.201 to 0.12), p=0.61	–0.005, p=0.95
	Externalizing	0.0088 (–0.021 to 0.07), p=0.62	–0.047 (–0.209 to 0.12), p=0.59	–0.038 (–0.198 to 0.12), p=0.69	–0.23, p=0.97
	Any	0.0139 (–0.0400 to 0.11), p=0.62	0.0297 (–0.198 to 0.25), p=0.85	0.0436 (–0.185 to 0.27), p=0.74	0.32, p=0.80
Gender	ADHD	4.4×10⁻⁶ (–0.013 to 0.02), p=0.97	–0.189 (–0.333 to –0.06), p<0.001	–0.189 (–0.328 to –0.06), p<0.001	–0.00002, p=0.97
	Externalizing	–0.0027 (–0.0391 to 0.03), p=0.846	–0.128 (–0.281 to 0.00), p=0.046	–0.131 (–0.280 to –0.01), p=0.038	0.02, p=0.848
	Any	–0.0046 (–0.0692 to 0.06), p=0.84	–0.150 (–0.343 to 0.05), p=0.13	–0.155 (–0.356 to 0.05), p=0.12	0.03, p=0.82
	Anxiety and Depression	–0.004 (–0.071 to 0.05), p=0.87	–0.077 (–0.299 to 0.13), p=0.44	–0.082 (–0.311 to 0.14), p=0.42	0.05, p=0.80
